# Daily social interactions related to daily performance on mobile cognitive tests among older adults

**DOI:** 10.1371/journal.pone.0256583

**Published:** 2021-08-26

**Authors:** Ruixue Zhaoyang, Stacey B. Scott, Lynn M. Martire, Martin J. Sliwinski

**Affiliations:** 1 Center for Healthy Aging, The Pennsylvania State University, University Park, PA, United States of America; 2 Department of Psychology, Stony Brook University, Stony Brook, NY, United States of America; 3 Human Development and Family Studies, The Pennsylvania State University, University Park, PA, United States of America; Max Planck Institute for Human Development, GERMANY

## Abstract

The lack of social contact or good social relationships has been linked with cognitive decline and higher risk for Alzheimer’s disease and related dementias. One important but unexamined question is how daily social interactions relate to older adults’ cognitive function in daily life. The present study examined how changes in daily social interactions related to fluctuations in older adults’ performance on mobile cognitive tests from day to day. Using an ecological momentary assessments approach, 312 older adults (aged 70 to 90 years) completed surveys on social interactions and mobile cognitive tests five times a day for 16 consecutive days using smartphones. Multilevel modeling was used for analyses. Results demonstrated that having more daily social interactions, especially more pleasant social interactions, related to better cognitive performance the same day and over the subsequent two days. Cognitive performance, however, did not predict subsequent changes in social interactions across days. At the between-person level, older adults who had more (*vs*. less) frequent interactions with close partners on average, especially with their friends, had better cognitive performance. Finally, the average levels of social interactions also moderated the within-person associations between daily social interactions and the same-day cognitive performance. In sum, results from this study highlight the importance of having pleasant social interactions and frequent interactions with friends for older adults’ cognitive function in daily life, and have important implications for future behavioral interventions targeting certain features of daily social interactions to reduce risk of cognitive decline and Alzheimer’s disease and related dementias.

## Introduction

As the worldwide population ages, Alzheimer’s disease is becoming a more common cause of death and one of the most expensive health conditions. Alzheimer’s disease and related dementias (ADRD) impose substantial burden on patients, their family and caregivers, and the economy [1]. Alzheimer’s disease is a progressive disease with a long preclinical phase in which pathophysiological processes begin years and even decades before the clinical symptoms or diagnosis [2]. Longitudinal studies suggest that detectable changes in cognitive function during the preclinical stage can predict the progression to the clinical stages of ADRD [2, 3]. Given the lack of effective pharmacologic treatments for ADRD, it is critical to identify malleable risk factors for cognitive decline as the targets of preventive interventions *prior to* the onset of the clinical stages of ADRD.

A growing body of evidence supports that the lack of social contact or positive social relationships is an important risk factor for cognitive decline and impairment, Alzheimer’s disease, and dementias (see [4–7] for reviews). For example, the 2020 *Lancet* Commission on dementia prevention, intervention and care identified social isolation at later life as one of the key risk factors for dementia, which accounted for comparable or higher percentage of dementia prevalence in the population than other well-established risk factors such as hypertension, depression, and physical inactivity [8]. However, previous research has mostly relied on global assessments of social relationships and lab-based neuropsychological tests of cognitive function. These global assessments were often administrated infrequently (at single or two time points) and thus were unable to capture the dynamic associations between individuals’ social experiences and cognitive function in daily life. In addition, although global measures of social relationships may provide useful information on individuals’ overall social connectedness, these measures were not designed to tap day-to-day social interaction experiences. Therefore, it is unclear how social interaction is related to fluctuations in cognitive function from day to day, and which type of daily social interaction is more beneficial for daily cognitive performance. To address these questions, the present study used ‘real time’ ecological momentary assessments (EMAs) in naturalistic settings to capture changes in both daily social interactions and performance in mobile cognitive tests among a sample of older adults, and further identify features of daily social interactions (e.g., frequency, quality, partner types) that best predict subsequent changes in cognitive performance in daily life. Findings from the current study have implications for interventions that target daily social interactions to improve cognitive function in later life.

An active and satisfying social life plays an important role in individuals’ health and well-being, as well as maintaining cognitive function at later life. Recent meta-analyses on longitudinal studies provide evidence to link different aspects of social interactions or social relationships with better cognitive function, including having a large social network, frequent social interactions, high levels of social engagement, good social relationships and social support [4–7]. Although the exact nature of the association between social interactions and cognitive function remains unclear, a variety of possible mechanisms have been proposed. According to the cognitive reserve hypothesis, engaging in social interactions, which are cognitively demanding, could provide cognitive stimulations and contribute to better cognitive outcomes via the building of cognitive reserve over time [9–11]. Social interactions may also enable people to receive social support from others, which could buffer against stress and its deleterious effects on cognitive outcomes [5, 12]. In addition, interacting with others, especially with one’s social network members, may encourage more positive health behaviors such as exercise and smoking cessation via social control and support, and thus benefit the cognitive health indirectly [13–15]. These mechanisms may act over different time spans, with their protective effects on cognitive function manifesting over days to years [16].

Little is known about how different features of daily social interactions relate to the fluctuations in cognitive function in everyday life. To our best knowledge, there are three studies that examined the link between daily activities, including social activities, and cognitive performance. Allard et al., (2014) examined the associations between daily experiences and mobile cognitive performance among older adults, and found that having socializing activities (telephone or in person) had no effect on mobile cognitive performance (semantic memory) over the subsequent three hours of the same day [17]. On the other hand, Bielak et al., (2017) found that on days when older adults engaged in more social activities, especially with close social partners, they had better performance on the memory and processing speed tests [16]. In another daily diary study, Neupert et al., (2006) found that on days when older adults experienced stressors, particularly interpersonal stressors with friends and family, they were more likely to report memory failures [18]. In sum, these studies provide some support for examining the associations between social interactions and cognitive performance at the daily level. However, none of these studies captured multiple features of social interactions and thus could not identify which features of daily social interactions are more beneficial for cognitive function. Also, none of these studies explored whether changes in cognitive function may predict subsequent social interactions. For example, declines in cognitive function may make it difficult for people to engage in and/or enjoy social interactions [19, 20]. Therefore, it is important to explore the reciprocal effects of cognitive performance on social interactions in order to disentangle the functions of social interactions as a risk factor or consequence of changes in cognitive performance.

### The present study

In light of these questions, the present study used an EMA approach to collect data on both social interactions and the objective performance on mobile cognitive tests in older adults’ natural environment. The coupling of temporally fine-grained, intensive assessments on social interactions with mobile cognitive tests enables us to examine the dynamic associations between daily social interactions and cognitive performance over different timescales. The first aim of the present study is to examine the within-person (WP) associations between distinct features of daily social interactions and performance on mobile cognitive tests within the same day and across days. Given that the frequency, quality and partner types of social interactions have been individually linked with health and cognitive function among older adults [16, 18, 20], the present study focused on these three features of daily social interactions and their relations with daily cognitive performance. In addition, we assessed individuals’ performance on several cognitive domains that have been linked with social experiences [5]: processing speed, attention, working memory and memory binding. We expected that higher frequency and better quality of social interactions would be related to better cognitive performance within and cross days *(H1&2)*, based on prior research on social relationships [4–7]. Past research also suggests that with increasing age, people prefer to interact with close social partners, such as family and friends, rather than with other peripheral partners [21]; and the interactions with close partners are more emotional meaningful and influential for older adults’ health and well-being [21, 22]. Therefore, we also expected that more frequent interactions with close partners, rather than other peripheral partners, would be related to better cognitive performance within and cross days *(H3)*. To test these hypotheses, we used time-lagged analyses to investigate different timescales over which the predictive effects of social interactions on cognitive function may manifest and persist (i.e., same-day and across days). As an exploratory extension, we also examined the reciprocal lagged effects of cognitive performance on social interactions over these timescales.

The second aim of the present study is to examine the between-person (BP) effects which address whether individuals who differed in their mean levels of social interactions would have better or worse cognitive performance on average, as compared with others. In addition, the present study tested the interactions between the WP and BP effects of daily social interactions in order to explore whether some individuals’ cognitive performance would be influenced more by their day-to-day social interactions as compared with others. Specifically, do individuals’ average levels of social interactions moderate the WP associations between daily social interactions and cognitive performance? Evidence from previous daily diary [23] and intervention studies [24, 25] suggest that the effects of certain activity (e.g., social, leisure or cognitive activity) may provide the greatest benefit for individuals who have more pronounced deficits in this type of activity. This is because there is more need or room for improvement among these individuals compared with those who already have high levels of activity. Therefore, we expected that the influences of day-to-day social interactions on cognitive performance would be stronger for individuals with lower (*vs*. higher) average levels of social interactions (*H4*). Knowledge gained from the current study is important to better understand the dynamic associations between daily social interactions and cognitive function, and also points to the best targets (e.g., certain features of social interactions, individuals with different social interactions patterns) and timing for effective behavioral interventions targeting daily social interactions to improve cognitive function.

## Materials and methods

### Participant and procedure

Data come from the ongoing Einstein Aging Study (EAS) and were collected between May 2017 and February 2020. Participants in EAS were recruited via systematic random sampling from Medicare and New York City Registered Voter Lists for Bronx County. The Albert Einstein College of Medicine Institutional Review Board (IRB) approved this study, and an IRB-approved form was used to obtain written informed consent from all participants. Screening was conducted by telephone to verify that prospective participants met inclusion criteria (English-speaking, community-residing, ambulatory individuals aged ≥ 70 years) and to enroll those who agreed to participate. Exclusion criteria included significant hearing or vision loss, current substance abuse, severe psychiatric symptoms that may interfere with testing, chronic medicinal use of opioids or glucocorticoids, treatment for cancer within the last 12 months, and a diagnosis of dementia. The final sample included 312 older adults (see Table 1 for descriptive information on the sample).

**Table 1:** Descriptive information on sample demographics and key study variables.

	Mean or %	SD
**Sample Demographics**		
Sex (female)	67.0%	
Age (years)	76.965	4.849
Race/Ethnicity		
White	45.5%	
Black	40.1%	
Hispanic	12.8%	
Other	1.6%	
College Degree or Higher	46.8%	
Currently Employed	9.0%	
**Study Variables** ^a^		
EMA Social Interactions		
Having any social interactions (yes)	77.7%	
Pleasant social interaction	84.0%	
Unpleasant social interaction	0.8%	
Ambivalent social interaction	4.2%	
Neutral social interaction	11.0%	
Interaction with only family members	37.2%	
Interaction with only friends	12.9%	
Interaction with only peripheral partners	12.3%	
Mobile Cognitive Tests		
Symbol Search ^b^	3295.290	1059.930
Grid Memory ^c^	2.290	1.308
Color Shape ^d^	0.614	0.414

Note: N = 312 persons for demographic information collected at the person-level; and n = 20,224 ecological momentary assessments (EMA) for social interactions and mobile cognitive tests.

^a^ Descriptive information on study variables were based on moment-level data from all EMA occasions in the study.

^b^ Unit: millisecond

^c^ Unit: Euclidean distance

^d^ Unit: Adjusted Proportion Correct (Hits-False Alarms).

Following the phone-screening assessment, eligible participants completed the consent process and were invited to attend a visit to the research clinic. During this visit, they completed questionnaires to assess demographics, psychosocial characteristics and cognitive status. Participants received 1.5 hours trainings of the EMA and mobile cognitive tests study protocol and were given one practice administration of each mobile cognitive test. The day following the clinic visit, participants began a 16-day EMA protocol, which involved six momentary assessments each day: a self-initiated wake-up assessment, a self-initiated end-of-day assessment, and four quasi-randomly prompted (i.e., beep) assessments. Beep assessments were spaced at approximately 3.5-hour intervals, varied in timing across days of the week (each day within a week has a unique beep schedule), and were programmed according to participants’ self-reported wake schedules. For each momentary assessment (e.g., wake-up, beep, or end-of-day), the participants answered self-report questions and performed the mobile cognitive tests at the end of each assessment using study-provided smartphones. The study-provided smartphones were locked so that the survey program was the only program on the phone that could be used by participants. Participants were also provided contact information in the event that they experienced any technological difficulties. After the 16-day EMA session, participants returned the study smartphones to the research clinic and completed more physiological and neurological exams. Participants received up to $160 as compensation. Because the self-report questions on social interactions were not included in the wake-up assessments, the current study only used data collected in beep and end-of-day assessments (5 assessments/day) on social interactions and mobile cognitive tests for analyses.

### Measures

#### EMA self-reports on social interactions

At each momentary assessment (beep or end-of-day), several questions were asked about social interactions that occurred since the last assessment (i.e., in the past 3 to 4 hours), capturing the frequency, quality, and partner types of social interactions. (1) **Frequency**: participants were at first asked whether they had any social interactions (defined as talking or spending time with someone in person, by phone/computer or by texting) since the last survey (1 = Yes, 0 = No). The answer “Yes” to this question across five surveys each day were summed up to indicate the frequency of having any types of social interactions that day (Range: 0–5). (2) **Quality**: if participants reported having any social interactions at each survey, they were then asked to categorize their most recent social interaction as “pleasant,” “unpleasant,” “neutral,” or “both pleasant and unpleasant.” The reported pleasant, unpleasant, neutral, or ambivalent (i.e., both pleasant and unpleasant) social interactions from five surveys each day were summed up respectively as indicators of the quality of daily social interactions. (3) **Partner Type**: if participants responded having any social interactions at each survey, they were also asked to select the partner(s) involved in the most recent social interaction from a list including spouse/partner, children, other family members, friends, neighbors, acquaintances, strangers, and others. On the basis of prior research [22], selected interaction partners were categorized into three different types: family (e.g., spouse/partner, children, other family member), friends, and other peripheral partners (e.g., acquaintances, neighbors, strangers and others). The frequency of social interactions with each of these three types of partners was calculated by counting the reported interactions with that type of partner only across all five surveys each day.

#### Mobile cognitive tests

**Fig 1:**
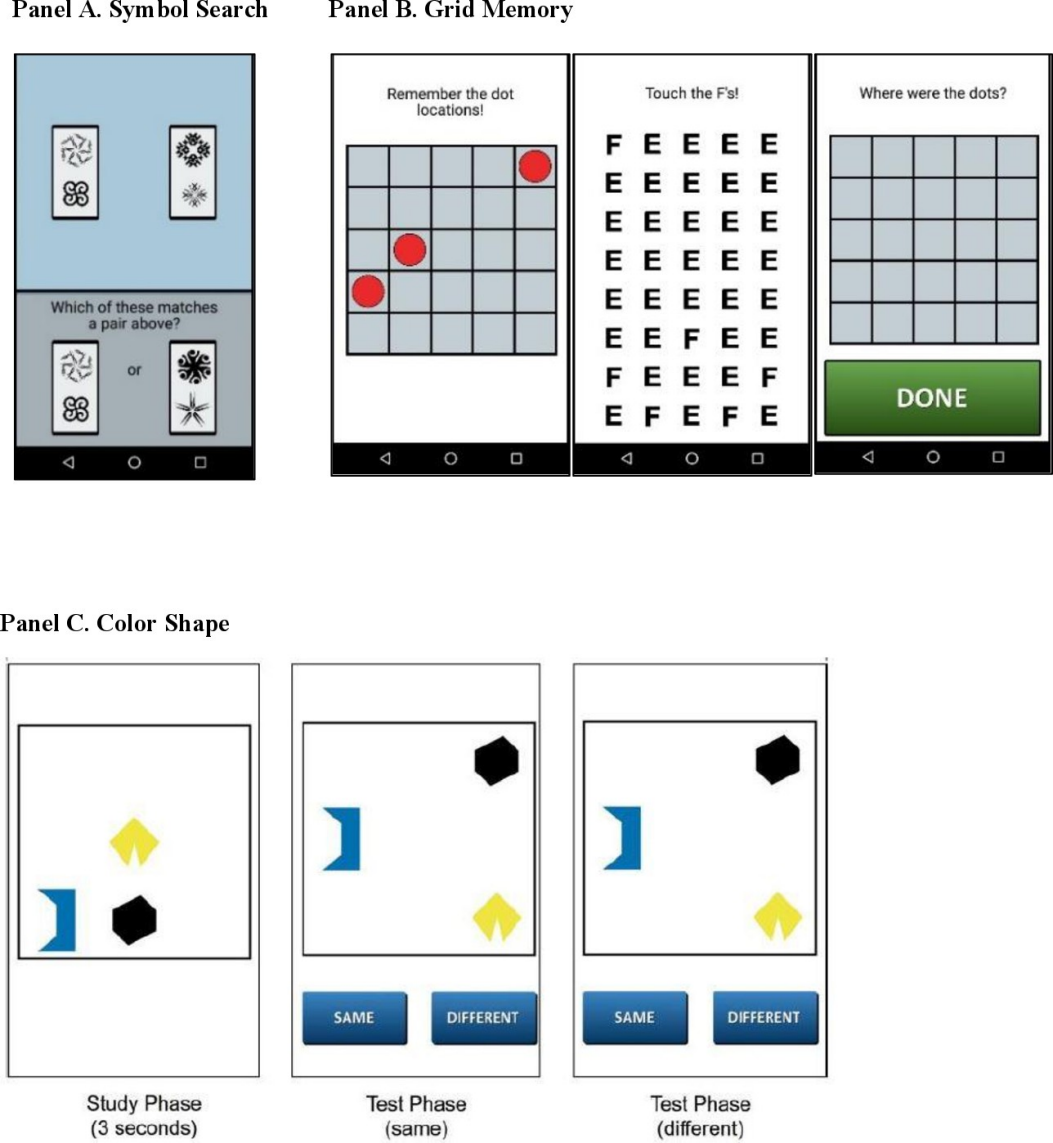
Mobile cognitive tests (panel A: symbol search; panel B: grid memory; panel C: color shape).

Participants completed the following mobile cognitive tests (Fig 1) at the end of each momentary assessment (beep or end-of-day):

**Symbol Search** (or Symbol Match, Fig 1, Panel A) is a cognitive test used to measure processing speed and attention [26, 27]. On each trial of the symbol search task, participants saw a row of two symbol pairs at the top of the screen and were presented with two symbol pairs at the bottom of the screen. Participants decided as quickly as possible which of the two pairs presented at the bottom of the screen was among the pairs at the top of the screen. Participants completed 11 trials of this task at each momentary assessment. The outcome variable was mean response time of correct trials and higher scores (longer response time) indicate worse performance.

**Grid Memory** (or Dot Memory, Fig 1, Panel B) is a free recall paradigm that assesses spatial working memory [27, 28]. This task involves a brief study (encoding) phase, during which 3 dots were presented at random locations on a 5 × 5 grid for 3 seconds, an 8-second letter-cancellation distraction phase, followed by free recall of locations occupied by dots during the study phase. The free recall phase required participants to touch the locations in an empty 5 × 5 grid that the 3 dots were presented initially. Participants completed 2 trials at each momentary assessment. The outcome variable was a mean score of Euclidean distance error scores from the 2 trials, which gives partial credit based on the deviation of the recalled compared to the correct dot locations. Higher scores indicate larger errors and thus worse performance on this test.

**Color Shape** (Fig 1, Panel C) is a change detection paradigm used to assess intra-item feature memory binding, which is the function that supports the integration of multiple elements or features of complex events [29, 30]. Three polygons of different shapes and colors were displayed for 3 seconds, then removed from the screen and re-displayed at different locations and either having the same or different colors. Participants completed 5 trials at each momentary assessment. The outcome variable was a recall score calculated as percentage of hits minus percentage of false alarms. Higher scores indicate better performance on this test.

### Statistical analysis

Analyses were conducted using multilevel modeling in SAS PROC MIXED with restricted maximum likelihood (REML) to address missing data [31]. The data from momentary surveys were aggregated at the day level (level 1), which were then nested within persons (level 2). Thus, social interactions and cognitive performance could vary over days within a person as well as across persons. All models included a random intercept to allow the mean scores of the outcome to vary across individuals. All level 1 predictors were person-mean centered and level-2 continuous predictors were grand-mean centered.

Four set of analyses were conducted. First, we examined the same-day within-person (WP) associations between daily social interactions and cognitive performance, controlling for the between-person (BP) effects of the average levels of social interactions on mean levels of cognitive performance (Model 1). Models were fit for each of the three mobile cognitive tests (as the outcome) separately and included a level 1 predictor of each feature of social interactions (frequency, quality or partner types), which captures each person’s daily deviation from his or her own mean score of social interactions (WP effect), and a level 2 predictor of the mean score of the same feature of social interactions which captures the individual differences in the average levels of social interactions (BP effect). Second, we examined the persistence of the predictive effects of daily social interactions on cognitive performance by exploring the lagged effects of social interactions across different days (e.g., 1, 2, 3, 4…*n* days). Only the results from the one-day and two-day lagged effects were presented because the majority of the lagged effects only extended over two days. Specifically, models were fit by predicting each of the mobile cognitive tests completed on day *t* from social interactions reported on day *(t—1)* (i.e., one-day lagged effect models, Model 2), or social interactions reported on day *(t—2)* (i.e., two-day lagged effect models, Model 3). In order to differentiate and account for the concurrent effect, social interactions occurred on day *t* were included in the one-day lagged effect models; and social interactions occurred on both day *t* and day *(t-1)* were included in the two-day lagged effect models. For exploratory analyses, we also examined the reciprocal lagged effects of cognitive performance on subsequent social interactions across day. Because the social interaction outcomes are count variables, multilevel Poisson models were used to predict day *t* social interactions from day *(t-1)* and day *(t-2)* cognitive performance, respectively.

Finally, we examined whether individual differences in the average levels of social interactions moderated the WP effects of daily social interactions on daily cognitive performance. Specifically, the cross-level interaction terms between the level 1 predictor of daily social interaction feature and the level 2 predictor of the person-level mean of the same social interaction feature were added to the same-day and cross-day models. All models above included the person-level covariates of sex (0 = male, 1 = female), age (years), and education (college degree or higher = 1; less than a college degree = 0), which have been linked with cognitive functions in the previous studies [8, 32, 33], as well as the day-level linear and quadratic trends (i.e., study day and study day^2^) to account for the retest-practice effects of cognitive tests.

## Results

### Descriptive information

The 312 participants in our sample completed 15.60 days of EMAs (*SD* = 1.49; *Range* = 2 to 16 days) on average, and provided valid data on social interactions and mobile cognitive tests in 16,156 beep and 4,068 end-of-day assessments. Participants reported having social interactions on 77.7% of all completed EMA surveys. The majority (84%) of reported social interactions were rated as “pleasant”, 11% as “neutral”, 4.2% as “both pleasant and unpleasant” (i.e., ambivalent), and only 0.8% interactions were rated as “unpleasant”. In terms of social interaction partners, 37.2% of all reported interactions involved only family members, 12.9% involved only friends and 12.3% involved only peripheral partners such as acquaintances, neighbors or strangers, whereas 37.6% of interactions involved more than one types of partners.

Aggregated scores for each of the mobile cognitive tests across the 16-day assessment period are also displayed in Table 1. All three cognitive tests (Symbol Search, Grid Memory, Color Shape) have excellent between-person reliability (*αs* > 0.98) [27, 34] and close to normal distributions (skewness < 0.9 for all three tests). All cognitive tests demonstrated significant retest-practice effects across the 16-day study period (*ps* < .000 for the linear trend; *ps* < .004 for the quadratic trend), with the performance improving quickly and considerably over the first two days and improving gradually afterwards.

### How were daily social interactions related to cognitive performance on the same day and across days (the WP effects)?

#### Same-day effects

A series of multilevel models were tested to examine the WP effects of three features of daily social interactions (frequency, quality, and partner types) on each of the three mobile cognitive tests conducted throughout the same day (summarized in Table 2, M1). As shown, the frequency of having pleasant social interactions one day was associated with better performance on both processing speed (Symbol Search, *b* = -11.422, *p* = .036) and memory binding (Color Shape, *b* = 0.005, *p* = .035) the same day, but was unrelated to performance on spatial working memory (Grid Memory). That is, on days when older adults had more pleasant social interactions than their own daily average, they had better performance on processing speed and memory binding tests that day. In addition, the frequency of interacting with family members was associated with better performance on memory binding test (Color Shape, *b* = 0.008, *p* = .005) the same day. That is, on days when older adults had more interactions with their family members (e.g., partner, kids, other family members) than their own daily average, they also performed better on memory binding test that day. The frequency of having any social interactions, having unpleasant, ambivalent (both pleasant and unpleasant), or neutral social interactions, or having interactions with friends or other peripheral partners did not relate to performance on any cognitive tests the same day.

**Table 2:** Summary of the same-day and cross-day within-person effects of daily social interactions on daily cognitive performance.

	Outcome = Day *t* Cognitive Performance								
	Symbol Search			Grid Memory			Color Shape		
Predictors: Features of Social Interactions (SI)	*Est*.	*SE*	*p*	*Est*.	*SE*	*p*	*Est*.	*SE*	*p*
***Frequency of SI***									
M1: Freq. of day *t* SI	-10.016	5.255	0.057	-0.002	0.008	0.761	0.005	0.002	0.058
M2: Freq. of day *t-1* SI	-15.439**	4.985	0.002	-0.010	0.008	0.206	0.004	0.002	0.113
M3 Freq. of day *t-2* SI	-14.036**	5.028	0.005	0.007	0.008	0.375	-0.001	0.002	0.710
***Quality of SI***									
M1: Freq. of day t pleasant SI	-11.422*	5.432	0.036	-0.005	0.008	0.562	0.005*	0.003	0.035
Freq. of day t unpleasant SI	-5.150	32.369	0.874	0.018	0.048	0.702	-0.001	0.015	0.934
Freq. of day t ambivalent SI	-11.340	14.961	0.449	-0.008	0.022	0.733	0.001	0.007	0.911
Freq. of day t neutral SI	3.251	10.305	0.752	0.017	0.015	0.276	0.001	0.005	0.893
M2: Freq. of day *t-1* pleasant SI	-14.479**	5.155	0.005	-0.012	0.008	0.138	0.005*	0.002	0.037
Freq. of day *t-1* unpleasant SI	-14.372	30.688	0.640	0.031	0.049	0.527	0.000	0.015	0.978
Freq. of day *t-1* ambivalent SI	-17.727	14.465	0.221	0.001	0.023	0.953	-0.003	0.007	0.618
Freq. of day *t-1* neutral SI	-20.639*	9.816	0.036	0.003	0.016	0.841	-0.003	0.005	0.462
M3: Freq. of day *t-2* pleasant SI	-13.744**	5.195	0.008	0.008	0.008	0.320	-0.001	0.003	0.658
Freq. of day *t-2* unpleasant SI	-38.342	31.974	0.231	-0.027	0.052	0.603	0.006	0.015	0.677
Freq. of day *t-2* ambivalent SI	-5.887	14.592	0.687	-0.030	0.024	0.210	-0.002	0.007	0.747
Freq. of day *t-2* neutral SI	-18.069	9.917	0.069	0.013	0.016	0.412	0.000	0.005	0.937
***Partner Type of SI***									
M1: Freq. of day t SI with family	-2.734	5.946	0.646	-0.008	0.009	0.392	0.008**	0.003	0.005
Freq. of day t SI with friends	-17.193	9.045	0.057	0.014	0.013	0.297	0.002	0.004	0.671
Freq. of day t SI with others	-8.811	9.057	0.331	0.008	0.013	0.542	-0.006	0.004	0.163
M2: Freq. of day *t-1* SI with family	-9.647	5.669	0.089	-0.012	0.009	0.184	0.002	0.003	0.520
Freq. of day *t-1* SI with friends	-10.451	8.630	0.226	0.002	0.014	0.904	0.004	0.004	0.332
Freq. of day *t-1* SI with others	-26.946**	8.692	0.002	0.016	0.014	0.236	0.002	0.004	0.618
M3: Freq. of day *t-2* SI with family	1.294	5.704	0.821	-0.003	0.009	0.754	0.001	0.003	0.792
Freq. of day *t-2* SI with friends	-7.383	8.780	0.400	0.003	0.014	0.806	-0.007	0.004	0.084
Freq. of day *t* -2SI with others	-8.562	8.745	0.328	0.003	0.014	0.851	-0.004	0.004	0.326

*Note*. N = 312 persons, n = 4190~4868 daily records. M1 = Model 1, same-day model; M2 = Model 2, 1-day lagged model; M3 = Model 3, 2-day lagged model. All listed effects are within-person effects. All models included the following covariates: Sex (0 = male, 1 = female), Age (years), Education (0 = no college degree, 1 = college or higher degree), Linear trend (study day), Quadratic trend (study day^2^). The BP effects of social interactions were also controlled in all models but were not listed; day t social interactions were controlled in M2; day t and t-1 social interactions were also controlled in M3 (not listed).

*p < .05.

**p < .01.

#### Cross-day lagged effects

Time**-**lagged models were used to examine the persistence of the WP effects of social interactions on cognitive performance across different days (e.g., 1, 2, 3…days), but only the one-day and two-day lagged effects were presented (M2 and M3 in Table 2) due to the non-significance of the majority of lagged effects beyond two days. As shown, having more than usual any types of social interactions one day ago (*b* = -15.439, *p* = .002) and two days ago (*b* = -14.036, *p* = .005) both predicted better performance on processing speed (Symbol Search) today. In other words, the predictive effects of having more social interactions on better processing speed manifested one day after the social interactions and persisted for two days (day 2 and day 3).

Similarly, having more than usual pleasant social interactions one day ago (*b* = -14.479, *p* = .005) and two days ago (*b* = -13.744, *p* = .008) both predicted better performance on processing speed (Symbol Search) today. In addition, having more than usual pleasant social interactions one day ago (*b* = 0.005, *p* = .037) also predicted better performance on memory binding test (Color Shape) today. Therefore, the predictive effects of having more pleasant social interactions on better cognitive performance extended over two days for processing speed test and over one day for memory binding test.

Furthermore, having more than usual neutral social interactions one day ago (*b* = -20.639, *p* = .036), or interactions with peripheral partners one day ago (*b* = -26.946, *p* = .002) also predicted better performance on processing speed today. No significant cross-day WP effects of any features of social interactions were found for Grid Memory test.

As mentioned, we also conducted analyses to explore reciprocal lagged effects of cognitive performance on subsequent social interactions across days. These analyses provided no strong evidence to support the lagged effects of cognitive performance on social interactions (See S1 Table for the results from one-day lagged analyses and S2 Table for results from two-day lagged analyses).

### How were average levels of daily social interactions related to average levels of cognitive performance (BP effects)?

Results of the BP effects from multilevel models (M1) were summarize in Table 3. As shown, the most robust finding is regarding daily social interactions with friends: On average, older adults who had more frequent daily interactions with their friends on average had better performance on all mobile cognitive tests—Symbol Search (*b* = -314.670, *p* = .004), Grid Memory (*b* = -0.185, *p* = .036), and Color Shape (*b* = 0.120, *p* = .000)—than those who interacted less frequently with friends. In addition, those who had more frequent interactions with family on average than others also performed better on memory binding test (Color Shape, *b* = 0.046, *p* = .012). Finally, the average levels of having any types of social interactions (*b* = -128.950, *p* = .009) and having pleasant social interactions (*b* = -127.020, *p* = .010) were also related to better performance on processing speed (Symbol Search).

**Table 3:** Summary of the between-person effects of daily social interactions on daily cognitive performance.

	Outcome = Day *t* Cognitive Performance								
	Symbol Search			Grid Memory			Color Shape		
Predictors: Features of Social Interactions (SI)	*Est*.	*SE*	*p*	*Est*.	*SE*	*p*	*Est*.	*SE*	*p*
***Frequency of SI***									
Average freq. of daily SI	-128.950**	49.087	0.009	-0.069	0.040	0.085	0.026	0.015	0.078
***Quality of SI***									
Average freq. of daily pleasant SI	-127.020*	49.542	0.010	-0.069	0.040	0.086	0.027	0.015	0.078
Average freq. of daily unpleasant SI	-104.930	682.440	0.878	0.056	0.556	0.919	-0.135	0.209	0.518
Average freq. of daily ambivalent SI	-6.312	185.240	0.973	0.160	0.151	0.290	-0.010	0.057	0.867
Average freq. of daily neutral SI	-188.200	111.980	0.093	-0.137	0.091	0.132	0.038	0.034	0.261
***Partner Type of SI***									
Average freq. of daily SI with family	-108.340	60.254	0.072	-0.032	0.049	0.516	0.046*	0.018	0.012
Average freq. of daily SI with friends	-314.670**	107.670	0.004	-0.185*	0.088	0.036	0.120***	0.032	0.000
Average freq. of daily SI with others	-110.060	124.300	0.376	-0.090	0.102	0.377	0.047	0.037	0.212

*Note*. N = 312 persons, n = 4868 daily records. All listed effects were between-person effects from the same-day models (M1 in Table 2). All models included the same day within-person effects of social interactions and the following covariates: Sex (0 = male, 1 = female), Age (years), Education (0 = no college degree, 1 = college or higher degree), Linear trend (study day), Quadratic trend (study day^2^).

*p < .05

**p < .01

***p < .001.

### Interactions between WP and BP effects

The cross-level interactions between the WP and BP effects of the same feature of social interactions were added into the models to predict the same-day as well as cross-day cognitive performance. The results indicated that the WP effects of having any social interactions, pleasant social interactions, or interactions with family members on the same-day performance on processing speed (Symbol Search) were all significantly moderated by the average levels of the same feature of social interactions across the 16-day study period. The interaction effect is *b* = 11.705, *p* = .033 for having any social interaction, *b* = 17.492, *p* = .001 for having pleasant social interactions, and *b* = 14.977, *p* = .021 for interacting with family members.

Simple slope tests revealed similar patterns across these three features of social interactions and supported our hypothesis. Specifically, having more than usual any social interactions (*b* = -21.933, *p* = .004), pleasant social interactions (*b* = -33.313, *p* < .000), or interactions with family members (*b* = -25.868, *p* = .026) was significantly associated with better performance on processing speed tests on the same day for older adults who on average had *less frequent* (one standard deviation below the sample mean) any interactions, pleasant interactions or interactions with family in daily life. In contrast, the WP effect of having more than usual any social interactions (*b* = 2.413, *p* = .758), pleasant social interactions (*b* = 6.918, *p* = .366), or interactions with family members (*b* = 4.085, *p* = .539) on the same day processing speed test was not significant for those who on average had *more frequent* (one standard deviation above the sample mean) that type of social interactions. In other words, the beneficial effects of having more than usual social interaction experiences (any type, pleasant, or interacting with family) on same-day processing speed were more pronounced for those who had lower (*vs*. higher) average levels of that type of social interactions in daily life. The WP effects of social interactions (same-day and cross-day) on performance on Grid Memory or Color Shape were not significantly moderated by the BP effects of the social interactions.

## Discussion

Social interaction in daily life is important for older adults’ health, well-being and cognitive function at later life. The present study is the first to examine how different features of daily social interactions—frequency, quality, and partner types—related to the fluctuations in older adults’ cognitive performance objectively assessed by multiple mobile cognitive tests at real-time and in their natural environment. Overall, this study revealed three primary findings. First, having more frequent daily social interactions, especially more frequent daily pleasant social interactions, related to better cognitive performance (processing speed and memory bindings) on the same day as well as over the next two days (WP effects). In contrast, performance on mobile cognitive tests did not predict subsequent changes in social interactions across days. Second, older adults who on average had more frequent interactions with close partners, especially with their friends, had better performance on mobile cognitive tests on average than those who had less frequent interactions with close partners (BP effects). Third, older adults who were relatively lacking in certain social interaction experiences (e.g., frequent interactions, pleasant interactions, or interactions with family) in general showed better cognitive performance (processing speed) on days when they had more than usual that type of social interaction experience (the WP and BP interactive effects). These findings provide strong evidence to support the role of daily social interactions as predictors for subsequent changes in daily cognitive function (processing speed, work memory, short-term memory binding) for older adults, and highlight the importance of having frequent, pleasant social interactions and frequent interactions with close partners for cognitive health in later life.

**Fig 2:**
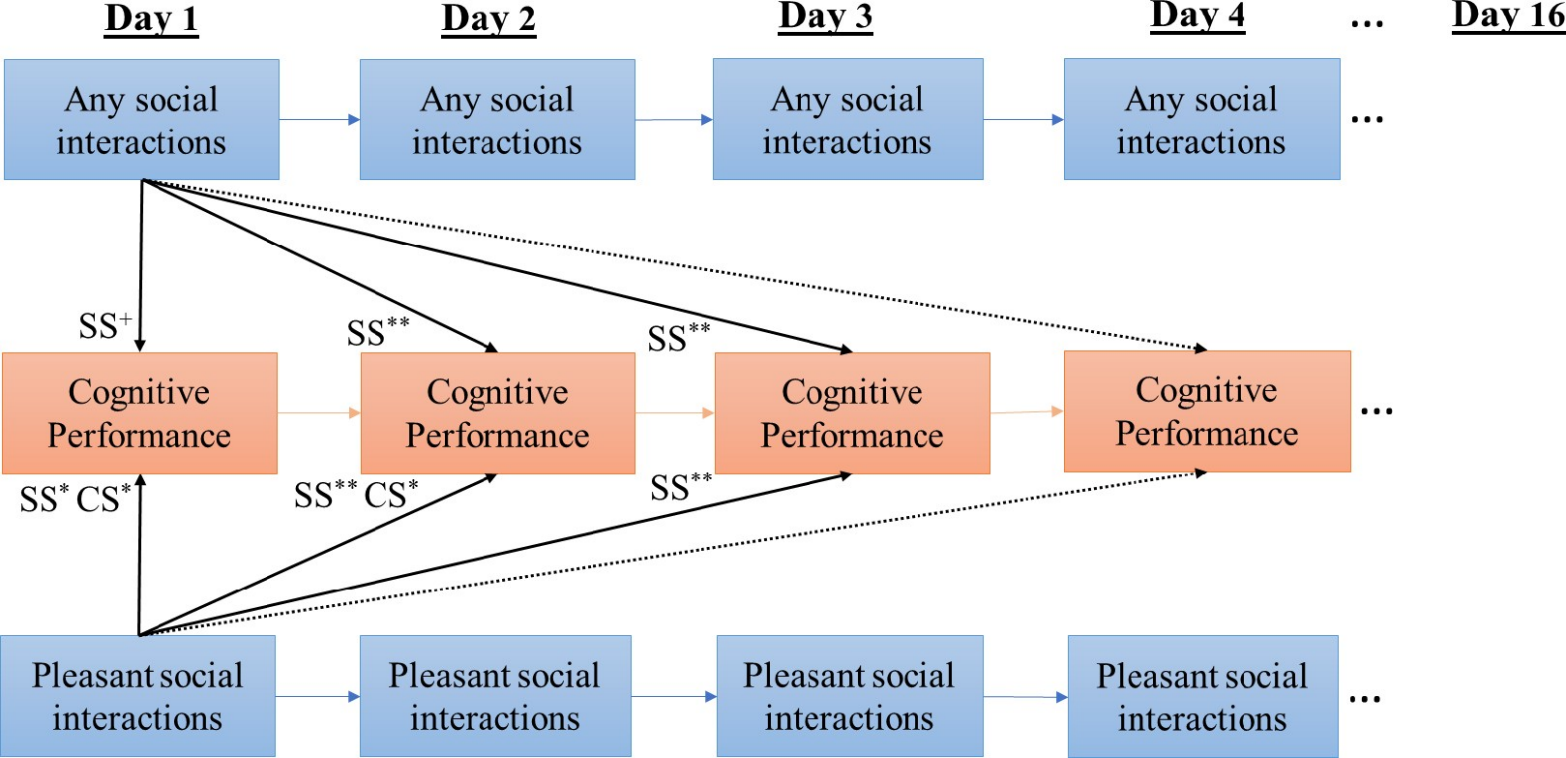
Summary of the within-person effects of daily social interactions on cognitive performance within the same day and across days (SS = Symbol Search; CS = Color Shape). Solid lines indicate significant effects and dashed lines indicate non-significant effects. ^+^ p < .10. * p < .05. **p < .01).

This study found evidence to support the hypotheses that higher frequency and better quality of social interactions related to better cognitive performance within and cross days *(H1&2)*. As summarized in Fig 2, significant WP associations were found between having frequent any type of or pleasant social interactions and the performance on mobile cognitive tests within the same day and across days. Particularly, on days when older adults had more than usual pleasant social interactions, they also had faster processing speed and better performance on the memory binding test on the same day as well as over the next one or two days. In contrast, evidence was not found to support the predictive effects of cognitive performance on subsequent changes in social interactions. These analyses provided strong evidence to support the enduring prospective effects of social interactions on subsequent cognitive performance, which is a critical first step toward establishing the causal relationship between daily social interactions and cognitive function. These findings join the growing body of evidence that being socially integrated and having good social relationships are important predictors for better cognitive functions and lower risks for cognitive decline and ADRD in later life [4–7].

The between-person findings that frequent interactions with family and friends, especially friends, are associated with better performance on cognitive tests on average are consistent with our hypothesis (*H3*) and previous research. For example, larger social networks with family and friends were associated with better global cognition over 5 years of followup [35]. Older adults with larger friend social networks were also found to have better memory scores than those with small friend networks [36]. Our study adds to previous research by showing that older adults who interact more frequently with family and friends in daily life in general also have better cognitive performance across multiple mobile cognitive tests, relative to those with less frequent interactions with family or friends in general. The interactions with peripheral partners such as acquaintance or stranger, however, were not consistently related to cognitive performance. It is possible that the interactions with emotionally close partners (*vs*. peripheral partners) are more likely to provide social support which could buffer against stress and its detrimental influences on cognitive health. The stronger social support and social control from family and friends (*vs*. peripheral partners) may also facilitate more health promoting behaviors, such as physical activity, which may in turn have beneficial sequelae for cognitive health.

Our study also extended previous research by demonstrating that some people may benefit more from changes in their daily social interactions than others. The WP associations between daily social interactions and processing speed test on the same day were stronger for those who had lower (*vs*. higher) average levels of social interactions in general. This pattern was found consistent across several features of daily social interactions—the frequency, quality (pleasant interaction) and partner types (family). These findings highlight the importance of considering individual differences to better understand the dynamic associations between daily social interactions and cognitive function.

Findings from this study also have important clinical implications for future behavioral interventions. Specifically, our results provide empirical evidence to support behavioral interventions that target daily social interactions as risk factors to improve cognitive health and reduce future risks for cognitive decline and ADRD. Using novel mobile technology, older adults’ daily social interactions and cognitive function could be closely monitored and enhanced by just-in-time adaptive interventions [37], which are designed to deliver highly personalized treatments in real time and in one’s natural environment [38, 39]. Our time-lagged analyses demonstrated the timescales over which the predictive effects of different features of social interactions on cognitive performance may manifest and persist, thus pointing out the best time windows for effective behavioral interventions targeting these features of social interactions. Furthermore, our findings regarding the interactions between the WP and BP effects of social interactions further point out that older adults who are relatively more deprived in certain social interaction experiences could potentially benefit the most from interventions that help to “boost” their usual levels of social interactions in daily life.

### Limitations and future directions

There are several limitations of this study that present promising avenues for future research. First, our study defined social interactions as talking or spending time with someone in person, by phone/computer or by texting, but did not clearly capture the channel of each reported social interaction episode (e.g., in person, telephone, or online). It is possible that in-person social interactions would have different effects on cognitive functions compared with online or telephone interactions. Future research is needed to further examine whether the associations between daily social interactions and cognitive function differ across distinct social interaction channels and whether social interactions via certain channel will benefit some individuals more based on their personal characteristics (e.g., age, gender, education, personality) or preference. Second, our study demonstrated the predictive effects of daily social interactions on cognitive performance over the short term (across days), and longitudinal data are needed to examine the effects of social interactions over longer terms (years). Finally, although this study did not find evidence to support the predictive effects of cognitive performance on subsequent changes in social interactions at the daily level, it is possible that the influence of declined cognitive performance on social interactions would demonstrate over longer time periods such as over years. Future research with data from both short- and long-term, such as measurement burst design across years, would provide opportunities to examine both the day-to-day and longitudinal bidirectional associations between social interactions and cognitive function.

## Conclusions

This study provided strong evidence to support the role of daily social interactions in cognitive health for older adults. Particularly, having frequent and pleasant social interactions was associated with improvement in performance on mobile cognitive tests over days. Older adults who had frequent interactions with friends in general also had better cognitive performance on average than others. These findings improve our understanding of the dynamic associations between daily social interactions and cognitive function as they unfold over time in daily life, and help to identify the specific features of daily social interactions as risk factors for future cognitive decline. The demonstration of the unique role of social interactions in cognitive function will pave the way for behavioral interventions targeting specific aspects of social interactions in daily life to reduce risk of cognitive impairment and ADRD.

## Supporting information

S1 Table Summary of the one-day lagged effects of daily cognitive performance on daily social interactions. (DOCX)

S2 Table Summary of the two-day lagged effects of daily cognitive performance on daily social interactions. (DOCX)
