# Supplementary material for: Daily social interactions related to daily performance on mobile cognitive tests among older adults
Source: PLoS One. 2021 Aug 26;16(8):e0256583. doi: 10.1371/journal.pone.0256583 (PMC8389411; doi:10.1371/journal.pone.0256583)
Supplement: S2 Table — (DOCX) [file pone.0256583.s002.docx]

S2 Table. Summary of the Two-Day Lagged Effects of Daily Cognitive Performance on Daily Social Interactions.

|  | **Predictor =Day *t-2* Cognitive Performance** | | | | | | | | | | | | | | | | | | |  |  |
| --- | --- | --- | --- | --- | --- | --- | --- | --- | --- | --- | --- | --- | --- | --- | --- | --- | --- | --- | --- | --- | --- |
|  | **Symbol Search** | | |  | **Grid Memory** | | | | | |  | | **Color Shape** | | | | | | |  |  |
| **Outcomes:**  **Day t Social Interactions (SI)** | *Est.* | *SE* | *p* |  | *Est.* | | *SE* | | *p* | |  | | *Est.* | *SE* | | | *p* | | |  |  |
|  |  |  |  |  |  | |  | |  | |  | |  | | |  | | |  | | |
| ***Frequency of SI*** | 0.006 | 0.025 | 0.798 |  | 0.002 | 0.016 | | 0.919 | |  | | -0.040 | | | 0.050 | | | 0.428 | | |  |
|  |  |  |  |  |  |  | |  | |  | |  | | |  | | |  | | |  |
| ***Quality of SI*** |  |  |  |  |  |  | |  | |  | |  | | |  | | |  | | |  |
| Freq. of pleasant SI | -0.006 | 0.027 | 0.816 |  | -0.005 | 0.017 | | 0.758 | |  | | -0.029 | | | 0.055 | | | 0.597 | | |  |
| Freq. of unpleasant SI | -0.108 | 0.281 | 0.702 |  | 0.234 | 0.183 | | 0.201 | |  | | -0.438 | | | 0.579 | | | 0.450 | | |  |
| Freq. of ambivalent SI | -0.046 | 0.120 | 0.700 |  | -0.029 | 0.078 | | 0.715 | |  | | 0.066 | | | 0.268 | | | 0.805 | | |  |
| Freq. of neutral SI | 0.121 | 0.074 | 0.102 |  | 0.008 | 0.050 | | 0.876 | |  | | -0.081 | | | 0.151 | | | 0.591 | | |  |
|  |  |  |  |  |  |  | |  | |  | |  | | |  | | |  | | |  |
| ***Partner Type of SI*** |  |  |  |  |  |  | |  | |  | |  | | |  | | |  | | |  |
| Freq. of SI with family | 0.024 | 0.040 | 0.550 |  | 0.023 | 0.026 | | 0.383 | |  | | -0.092 | | | 0.081 | | | 0.256 | | |  |
| Freq. of SI with friends | 0.021 | 0.072 | 0.767 |  | -0.078 | 0.044 | | 0.076 | |  | | -0.014 | | | 0.146 | | | 0.921 | | |  |
| Freq. of SI with others | 0.015 | 0.073 | 0.834 |  | 0.095 | 0.047 | | 0.046 | |  | | 0.132 | | | 0.156 | | | 0.398 | | |  |

*Note.* N=312 persons, n=4189 daily records. One multilevel Poisson model was estimated for each of the count outcomes (e.g., frequency of SI, freq. of pleasant SI, freq. of unpleasant SI). All listed effects are within-person effects. All models included the following covariates: Sex (0=male, 1=female), Age (years), Education (0=no college degree, 1=college or higher degree), Linear trend (study day), Quadratic trend (study day^2^), and day t and day t-1 cognitive performance. For model converge purposes, response times for Symbol Search were recoded to be in seconds.
